# Three-Dimensional Radiographic Evaluation of the Malar Bone Engagement Available for Ideal Zygomatic Implant Placement

**DOI:** 10.3390/mps3030052

**Published:** 2020-07-22

**Authors:** Gerardo Pellegrino, Francesco Grande, Agnese Ferri, Paolo Pisi, Maria Giovanna Gandolfi, Claudio Marchetti

**Affiliations:** 1 Oral and Maxillofacial Surgery Unit, Department of Biomedical and Neuromotor Sciences, University of Bologna, 40125 Bologna, Italy; agnese.ferri3@unibo.it (A.F.); Claudio.marchetti@unibo.it (C.M.); 2 Oral Surgery Unit, Dental School, Department of Biomedical and Neuromotor Sciences, University of Bologna, 40125 Bologna, Italy; francesco.grande6@unibo.it; 3 Dental Radiology Unit, Dental School, Department of Biomedical and Neuromotor Sciences, University of Bologna, 40125 Bologna, Italy; paolo.pisi@unibo.it; 4 Medical-technical Science, Dental School, Department of Biomedical and Neuromotor Sciences, University of Bologna, 40125 Bologna, Italy; mgiovanna.gandolfi@unibo.it

**Keywords:** malar bone, zygomatic implants, edentulism, implant path, prosthetic planning, implant planning, implant and maxillary sinus, skeletal landmarks, cone beam computed tomography

## Abstract

Zygomatic implant rehabilitation is a challenging procedure that requires an accurate prosthetic and implant plan. The aim of this study was to evaluate the malar bone available for three-dimensional zygomatic implant placement on the possible trajectories exhibiting optimal occlusal emergence. After a preliminary analysis on 30 computed tomography (CT) scans of dentate patients to identify the ideal implant emergencies, we used 80 CT scans of edentulous patients to create two sagittal planes representing the possible trajectories of the anterior and posterior zygomatic implants. These planes were rotated clockwise on the ideal emergence points and three different hypothetical implant trajectories per zygoma were drawn for each slice. Then, the engageable malar bone and intra- and extra-sinus paths were measured. It was possible to identify the ideal implant emergences via anatomical landmarks with a high predictability. Significant differences were evident between males and females, between implants featuring anterior and those featuring posterior emergences, and between the different trajectories. The use of internal trajectories provided better bone engagement but required a higher intra-sinus path. A significant association was found between higher intra-sinus paths and lower crestal bone heights.

## 1. Introduction

The modern trend in implantology is to reduce surgical invasiveness [1]. This reduces patient morbidity, costs, and complications [2]. Today, it is very often the case that short, tilted, or zygomatic implants replace bone grafts [3] when treating advanced cases of maxillary atrophy. Zygomatic implants seem to be a valid alternative to reconstructive procedures for severely atrophic maxilla [4,5,6].

On the other hand, zygomatic implant placement is a challenging procedure for oral and maxillofacial surgeons requiring the evaluation of many clinical, anatomical, and prosthetic parameters. The lack of soft and hard tissues caused by the extreme resorption of the upper maxilla requires an accurate preoperative planning in order to optimize the results and avoid complications. Careful radiographic analysis of bony topography with special attention to the orbital socket, infratemporal fossa, and maxillary sinus is essential before implant positioning [7]. The definition of the implant emergence position in the oral cavity, the amount of bone engagement, and the trajectory of the zygomatic fixtures are necessary for the safety of the patients and for a successful prosthetic rehabilitation.

This technique provides double implant anchorage within the malar bone body and (when possible) additional anchorage within the alveolar bone crest. Bicortical retention is the optimal form of prosthetic support [4]. The first zygomatic implant placement technique proposed by Brånemark [7] involved an intramaxillary sinus implant trajectory, usually associated with palatal implant emergence, which caused patient discomfort due to the excessive extension of the prosthesis. Nonmechanical problems included difficulty in maintaining peri-implant hygiene and pronunciation disorders in oral communication [8,9]. Thus, several modifications of this implant placement technique have been proposed by various authors [10,11]. An extra-sinus trajectory was used to place the implant platform in a more suitable position for prosthetic rehabilitation [12,13,14]. Indeed, in cases of extreme maxillary atrophy, the alveolar bone is not present due to severe bone resorption. This changes the amounts of malar and alveolar bone to which the implant can be fixed, and the implant trajectory can be influenced on the basis of the anatomy as the “zygoma anatomy guided approach” (ZAGA) [15]. According to the latest guidelines, zygomatic implant positioning should be prosthetically guided. The latest technology of static and dynamic surgery has already been used for standard implants [16,17] and has also been used for the zygomatic implants [18,19] to reproduce the preoperative plan. This requires preventive evaluation of implant platform emergence and implant trajectory.

Some authors [20,21,22] have performed extensive research regarding the extent of zygomatic bone in which implants can be inserted. However, the data are insufficient to evaluate the most adequate implant position and trajectory in order to engage the greatest amounts of malar bone. This is essential during preoperative planning of zygomatic implants as well as the choice of implant length. These procedures are often very difficult; the three-dimensional implant pathway is tilted on all the three spatial axes as the prosthetic abutment is angulated about 55 degrees, which makes it hard to use standard radiograph templates that can identify the implant emergences via standard planning software. Due to the cross-section drawing, the implant trajectory cannot coincide with the prosthetic axis of the abutment. Thus, some clinicians simulate the clinical procedure on stereolithographic models prior to surgery [18].

The aims of the present study were to measure the extent of malar bone engaged by zygomatic implants exhibiting optimal occlusal emergence and to define the available three-dimensional trajectories. The secondary objectives were to evaluate intra- and extra-sinus pathways and their associations with bone crest atrophy.

In order to find the ideal occlusal emergences for each of the four zygomatic implants on edentulous patients, we performed a cephalometric and skeletal analysis in CT scans of dentate patients and we reported the found landmarks in CT scans of edentulous patients.

## 2. Materials and Methods

The study featured two principal steps that were defined as phase 1 and phase 2.

The aim of phase 1 was to identify bilaterally, via cephalometric planes and skeletal landmarks, the position of lateral incisors/canines and second premolars/first molars in dentate patients. These positions correspond to the four ideal zygomatic implant emergence points for a quad approach [19] commonly used to treat totally edentulous patients with severe maxillary atrophy. Thus, these points found on the dentate patient were transferred onto the edentulous patient in order to identify the ideal implant emergence for a quad approach without the need of a diagnostic radiographic template.

Secondly, starting from the four ideal zygomatic implant emergence points, we measured the extent of malar bone engageable by zygomatic implants on the possible trajectories.

### 2.1. Phase 1

Thirty head and neck computed tomography (H&N CT) scans of dentate patients, who gave their consent, were collected from the Sant’Orsola University Hospital of Bologna database, complying with the Declaration of Helsinki principles. The data were recorded anonymously and scheduled with a patient ID number regarding only age and sex from the database. Only H&N CT exams prescribed for issues different from cranio-maxillo-facial deformities and tumors were included in the present retrospective study. The patients who did not have a complete denture at least up to the second molar were excluded. The CT data were evaluated using SimPlant O&O software. Three-dimensional skull models were created using the function “Create 3D objects-Segmentation wizard”. The models were created using thresholding and region-growing tools, reflecting the greyscale values of the pixels. Then, the porion, orbital, incisive foramen, and zygomaxillary points were identified; the latter are the most inferior points of the maxillary zygomatic suture [21]. Next, the “3D Cephalometry” function was used to define the Frankfurt horizontal plane passing through the porion and orbital points, constructing the first cephalometric plane (ImpB) normal to the Frankfurt plane and passing through the two zygomaxillary points: right and left (ZmR and ZmL).

**Figure 1:**
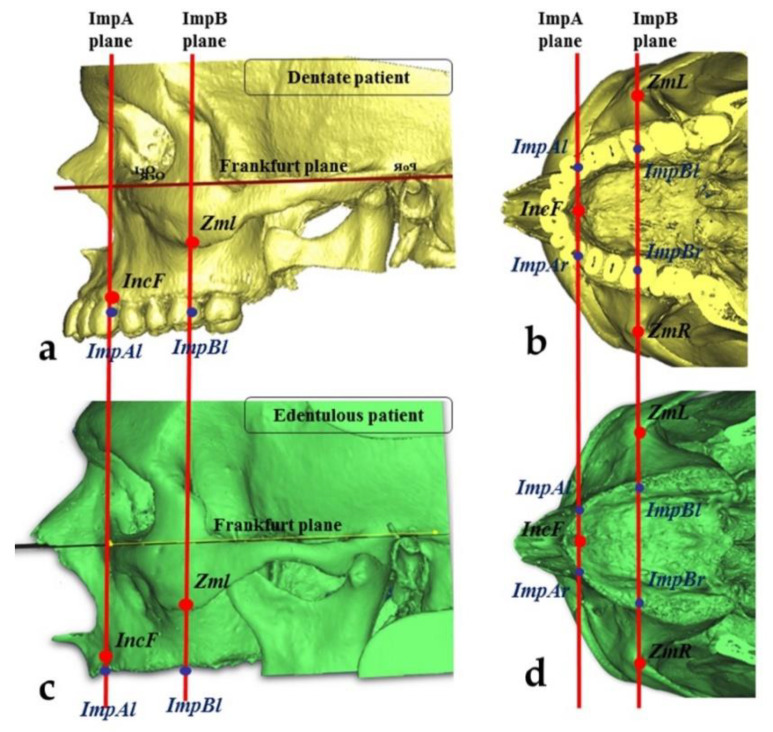
Identification of the ideal implant emergences corresponding to the positions of the lateral incisors/canines (for anterior zygomatic implants) and second premolars/first molars (for posterior implants). (**a**) Lateral view of a dentate patient; (**b**) occlusal view of a dentate patient; (**c**) lateral view of a edentulous patient; (**d**) occlusal view of a edentulous patient.

Then, another cephalometric plane (ImpA) parallel to ImpB and passing through the posterior part of the incisive foramen was defined. Finally, the dentate occlusal maxillary points in the ImpA and ImpB planes were fixed on both the right (ImpAr, ImpBr) and left (ImpAl, ImpBl) (Figure 1).

According to Branemark [7], Uchida [21], Rossi [23], Triplett [24] and the ideal emergence points of zygomatic implants placed using the four-zygoma technique lie near the lateral incisors/canines for anterior implants and near the second premolars/first molars for posterior implants. Then, the correspondences between the anterior implant points (ImpAr and ImpAl) and the lateral incisors/canines (the area between the mesial part of the lateral incisor and the distal part of the canine) were measured, as well as the correspondences between the posterior implant points (ImpBr and ImpBl) and the second premolars/first molars (the area between the mesial part of second premolar and the distal part of the first molar).

### 2.2. Phase 2

In the second step, the extent of malar bone engageable by zygomatic implants exhibiting ideal emergences were measured (at ImpAr/ImpBr or ImpAl/ImpBl) on the possible trajectories. The secondary outcomes were the evaluation of the intra-sinus pathway for each hypothetical trajectory and the extent of alveolar bone atrophy at the implant emergence positions. All measurements were performed by a blinded operator under the supervision of two experts in oral and maxillofacial surgery and radiology, similarly to Hung [22].

### 2.3. Measurements of the Zygomatic Bone

Eighty head and neck CT of patients with edentulous maxillae, having less than 5 mm of height present in the posterior area, were collected from Sant’Orsola University Hospital database as previously prescribed, and were analyzed using Materialise Mimics Innovation Suite 17.0 Medical. All the patients gave their consent, and all the procedures complied with the Declaration of Helsinki. The procedures described above were used to identify the ideal emergence points for edentulous patients. The two cephalometric planes (ImpA and ImpB) were constructed on three-dimensional skull models to locate the ideal implant emergence points on the alveolar bone (ImpAr/ImpBr or ImpAl/ImpBl) (Figure 1). Then, two other points in the zygomatic bone were identified:

- The “upper jugale”: the highest point of the posterior frontal process eminence of the zygomatic bone
- The posterior point of the frontozygomatic suture.
  Two planes were drawn using the “CAD Objects” tool:
  - The “zygomatic anterior” plane, showing the ideal emergence points of prosthetic anterior implants; these were ImpAr/ImpAl and the upper jugale.
  - The “zygomatic posterior” plane, showing the ideal emergence points of prosthetic posterior implants; these were ImpBr/ImpBl and the posterior point of the frontozygomatic suture.

**Figure 2:**
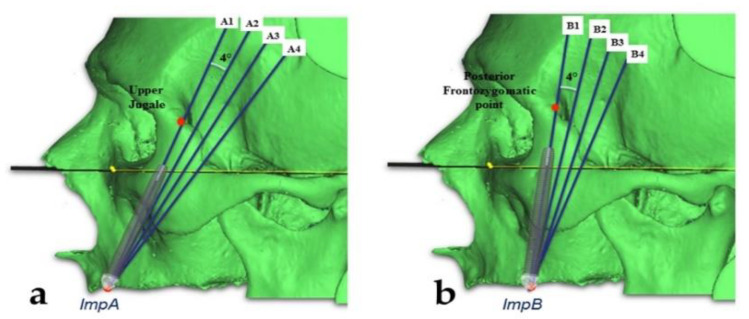
(**a**) Sagittal planes drawn clockwise, rotating by 0, 4, 8, and 12° from the upper jugale on the ImpA points for the anterior zygomatic implant trajectory. (**b**) Sagittal planes drawn clockwise, rotating by 0, 4, 8, and 12° from the posterior frontozygomatic point on the ImpB points for the posterior trajectory.

A cross-section slice of each plane was obtained using the function “Create reslice plane”. Each resliced object was rotated (using “Rotate”) in the sagittal plane (*x*-axis) in 4° steps, while holding the ideal occlusal points (ImpAl, ImpAr, ImpBl, and ImpBr) fixed. In this manner, eight different CT frontal slices (four for each plane) were obtained by rotating the zygomatic anterior and posterior planes through 0, 4, 8, and 12° angles (Figure 2).

**Figure 3:**
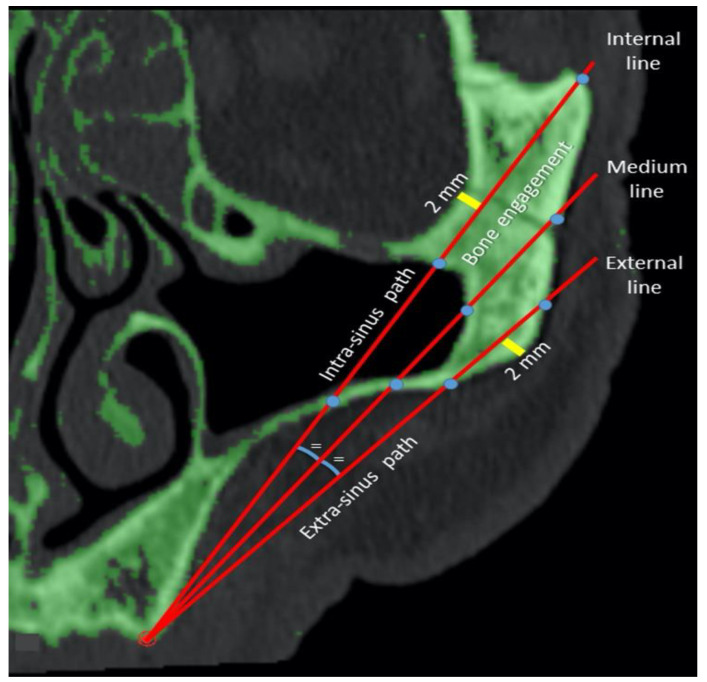
Measurements of the intra- and extra-sinus pathways and bone engagement on the internal, intermediate, and external possible trajectories. Lines 1, 2, and 3 are the internal, intermediate, and external trajectories respectively.

The two sides were considered separately. On each slice, the two zygoma were considered separately. Three different ideal implant trajectories (internal, intermediate, and external) were identified for each zygoma. Starting from each of the ideal occlusal points (ImpAl, ImpAr, ImpBl, and ImpBr), we drew the internal trajectory at 2 mm to the internal limit (the orbital cavity or the infratemporal fossa) and the external trajectory at 2 mm to the external cortical layer of malar bone. The intermediate implant trajectory was identified by bisecting the angle between the internal and external paths. We then measured the lengths of engaged malar bone (the implant length from the middle point of the bone crest to the external cortical point of the malar bone) and drew intra- and extra-sinus paths for each trajectory (Figure 3).

Differences among the trajectories, anterior/posterior emergences, sex, and age were evaluated. In addition, the extent of alveolar bone atrophy was evaluated at each implant site. The most external implant trajectories (those lying maximally outside the sinus) were classified using the ZAGA classification [15]. All data were compared using SPSS statistical software. Each dependent variable (total implant length, length of engagement with malar bone, and the intra- and extra-sinus path lengths) was subjected to univariate factorial analysis of variance. Multiple comparisons were performed using Tukey’s HSD test. Simple linear regression analysis was used to predict the relationships of age with length of malar bone engagement and total length. The significance level was set to 0.05.

## 3. Results

### 3.1. Phase 1

In all 30 cases of dentate patients, the cephalometric planes ImpA and ImpB crossed the ideal occlusal or prosthetic points, previously identified as the lateral incisors/canines for anterior zygomatic implants and the second premolars/first molars for posterior implants. Thus, the ideal prosthetic points were defined by reference to previously identified skeletal landmarks in order to accurately locate these points even in edentulous maxillae.

### 3.2. Phase 2

There were 47 male and 33 female patients, and the mean age was 79.4 years (range 49–101 years). The mean total lengths available for zygomatic implants, of malar bone engagements, and of the intra- and extra-sinus paths for different trajectories and rotation planes are summarized in Table 1 and Table 2.

The mean total length from implant emergence to the malar bone exit was 53.77 ± 9.33 mm for all trajectories. The mean malar bone engagement length was 17.91 ± 6.62 mm, and the lengths of the intra- and extra-sinus paths were 5.99 ± 8.91 and 29.86 ± 8.64 mm, respectively. The mean total length was 58.32 ± 7.44 mm for anterior emergence and 49.23 ± 8.81 mm for posterior emergence; the difference was statistically different (*p* = 0.000). In addition, the differences among the internal, intermediate, and external trajectories, and those among the rotation planes (0, 4, 8, and 12°), were statistically significant (*p* < 0.05). The upper line and the 4° plane exhibited the greatest lengths (61.82 ± 6.95 and 55 ± 9.99 mm, respectively). In males and females, the total lengths were 55. 13 ± 9. 42 and 51. 84 ± 8. 85 mm, respectively; the difference was statistically significant (*p* = 0.000).

In terms of malar bone engagement, the anterior emergence was significantly greater than the posterior emergence (19.55 ± 6.01 vs. 16.27 ± 6.80 mm). The internal line featured 20.03 ± 8.06 mm of bone engagement, which was significantly (*p* < 0.05) longer than those of the other trajectories (18.99 ± 6.30 and 14.72 ± 3.35 mm for the intermediate and external lines, respectively). The rotation plane affording maximal bone engagement was the 4° plane (19.42 ± 6.45 mm). The values for the other planes were 18.60 ± 6.18 mm (0°), 17.55 ± 6.64 mm (8°), and 16.09 ± 6.4 mm (12°). The differences were statistically significant. The difference between males and females was a borderline significance (*p* = 0.48) in favor of males (18.02 ± 6.76 vs. 17.76 ± 6.42 mm).

The internal line featured the greatest bone engagement, but also the greatest intra-sinus component (13.28 ± 10.16 mm); the intermediate line exhibited high-level engagement (18.99 ± 6.30 mm) but a significantly shorter intra-sinus path (4.65 ± 6.66 mm). The external line facilitated extra-sinus implant positioning, exhibiting the longest (*p* < 0.05) extra-sinus path (31.60 ± 6.29 mm) but the least bone engagement (14.72 ± 3.35 mm).

In terms of the emergence position, the 4° plane was associated with the highest engagement (19.42 ± 6.45 mm) but also the longest intra-sinus path (7.69 ± 9.96 mm). On the other hand, the 12° plane showed the lowest intra-sinus component and engagement (16.09 ± 6.74 mm). The 0° and 8° planes were intermediate, with bone engagements of 18.60 ± 6.18 and 17.55 ± 6.64 mm and intra-sinus paths of 6.32 ± 9.5 and 6.14 ± 8.73 mm, respectively.

Anterior emergence was associated with significantly (*p* = 0.000) greater engagement than was posterior emergence (means: 19.55 ± 6.01 and 16.27 ± 8.80 cm, respectively) and a larger extra-sinus component (33.9 ± 5.13 and 25.75 ± 9.44 mm, respectively). Notably, the greatest extents of bone engagement were evident in the upper lines of the 0 or 4° planes (24.26 ± 5.84 or 24.09 ± 6.36 mm, respectively), and the lowest intra-sinus components were associated with the lowest lines. When posterior emergence was in play, the optimal bone engagement was attained using an internal line following the 4° plane (23.08 ± 6.92 mm). The bone engagements and intra-sinus paths to anterior and posterior emergences employing different lines and planes are listed in Table 3.

Of the entire alveolar bone, 13.1% was < 2 mm in height, 13.4% was 2–4 mm, and 73.4% was >4 mm. In terms of alveolar bone height, a crest < 2 mm was associated with the longest intra-sinus path (9.37 ± 11.79 mm), and that of 2–4 mm or > 4 mm in height was associated with a path length of 7.39 ± 10.24 or 5.12 ± 7.81 mm, respectively; the difference was statistically significant (*p* = 0.049). Very weak correlations were evident between age and total length (R = 0.09) and between age and the extent of bone engagement (R = 0.04).

Of all trajectories, 2.8% were classified as ZAGA 0, 25.9% as ZAGA 1, 31.9% as ZAGA 2, and 39.4% as ZAGA 3. The ZAGA 3 trajectory featured the greatest bone engagement and the shortest intra-sinus path, whereas the ZAGA 1 trajectory was associated with the least bone engagement and the longest intra-sinus path.

## 4. Discussion

In this study, it was possible to identify the ideal emergences of both anterior and posterior zygomatic implants, according to data that had already been reported in the literature [21,22,23].

These anatomical points, identifiable via cephalometric planes based on a visible anatomic landmark, showed a high (100%) percentage of correspondence with the position of teeth that represent the ideal implant emergences (lateral incisors/canines and second premolars/first molars). The same anatomical landmarks (incisive foramen and zygomaxillary point) available for edentulous patients can be useful for the 3D planning, as they are clearly clinically identified during the surgery, intraoperatively.

The extents of malar bone available for zygomatic implants using various trajectories and the intra-/extra-sinus trajectory implications were evaluated. The data found can be used to facilitate preoperative three-dimensional virtual planning. The mean total length from implant emergence to the point of exit from the malar bone was 53.77 ± 9.33 mm (range 30.39–82.83 mm). Similar values have been reported in other studies. Uchida et al. [21] and Xu et al. [25] reported average lengths of 50.2 ± 4.13 mm (range 44.3–54.3 mm) and 58.15 ± 7.50 mm (range 36.46–72.35 mm), respectively. If anterior emergence was contemplated at the level of the canines/first premolars, the implant length required to attain the external cortical area of malar bone was 58.32 ± 7.44 mm. To maximize both engagement and the extra-sinus component, either the midline trajectory of the 8° plane (20.84 ± 5.87 mm of bone engagement and 4.52 ± 5.55 mm of an intra-sinus path) or the 0° plane (19.02 ± 4.93 mm of bone engagement and 3.28 ± 5.10 mm of an intra-sinus path) should be chosen. A posterior implant (length 49.23 ± 8.81 mm) emerging at the level of the second premolar/first molar is associated with less mean bone engagement compared with an anterior implant. However, a good compromise in terms of limiting sinus invasion would be to employ the midline of the 8° plane (18.40 ± 7.10 mm of bone engagement and 4.32 ± 6.93 mm of an intra-sinus path). Various implant trajectories can be chosen depending on the surgeon’s preference for an intra- or extra-sinus approach. Differences in zygoma implant intra-malar length by sector were reported by Bertos Quilez et al. [26] and Hung et al. [20]. Rossi et al. [23] also found a statistically significant difference between the mean length from the crestal point to the orbital socket in both the canine (53.42 ± 4.08 mm) and premolar (42.47 ± 3.20 mm) regions. Regarding anatomical and radiological studies on zygomatic bone, Nkenke et al. [27] found an average anterior–posterior length of the zygomatic bone of 25.40 ± 2.64 mm for female and of 24.93 ± 4.67 mm for male specimens, with a medium-lateral thickness of 7.60 ± 1.45 mm for females and 8.00 ± 2.26 mm for males. This is to assess the quantity of the zygomatic bone. In this study, the total length in males (59.59 ± 7.50 mm) was significantly higher than that in females (56.29 ± 6.85 mm). Hung et al. [22] found a statistically significant between-sex difference in terms of both the length and thickness of the zygomatic bone, while Takamaru et al. [28] (using a different method) did not. In the present study, the lengths in males and females were 55.13 ± 9.42 mm and 51.84 ± 8.85 mm, respectively, and this difference was statistically significant.

In this study, the total bone engagement was 17.92 ± 6.92 mm. Bertos Quilez et al. [26] reported a mean intra-malar length of 16.95 ± 4.73 mm. Balshi et al. [29] found a bone implant contact (BIC) of 15.5 ± 6.0 mm in men and 14.7 ± 5.4 mm in women. Hung et al. [22] reported a mean BIC of 16.70 ± 4.18 mm on the superior area of the zygoma. Corvello [30] did not use a CT-based method, but rather employed a probe to measure dry skulls, finding a mean BIC of 14.11 ± 5.93 mm using an exteriorized technique and 8.39 ± 2.9 mm using the intra-sinus Brånemark approach [7]. Similarly, in the present study, the extent of bone engagement was greater when the positioning was more internal with respect to the intra-sinus placement (19.3 ± 6.95 mm for the ZAGA 3 class but 14.65 ± 4.88 mm for the ZAGA 0 class). In terms of the ZAGA classifications, ZAGA 3 and 2 were the most common (39.4 and 31.9%). In all cases, 25.9% were ZAGA 1; we encountered no ZAGA 0 cases. Aparicio et al., who developed the ZAGA classification, reported rates of 15%, 49%, 20.5%, 9%, and 6.5% for ZAGA 0–4, respectively.

The most significative limitation of this study was the patient selection. In fact, the cases considered in the study were edentulous patients with advanced bone atrophy in the posterior maxilla. Some of these cases may be rehabilitated also with other techniques. Moreover this study presented no interindividual variability between several operators, as the measurements were performed by a single operator supervised by two experts in oral and maxillofacial surgery and radiology, similarly to Hung et al. [22]. Another limitation was the comparison with the other studies that did not evaluate the position of the implant emergence. The results of this study suggest that greater bone engagement can be obtained with a more internal implant trajectory than with an external trajectory, but the opposite was true in terms of the intra-sinus path length. The best angulation on the sagittal plane for bone engagement was 0 or 4°. The intra-sinus path length increased when the bone crest was <2 mm. The data can be considered to facilitate the three-dimensional planning of zygomatic implants. A clinical implication of what was found is the opportunity to use anatomical landmarks, usable also during surgery, instead of a standard template for surgical planning. A standard prosthetic radiographic template is not always used for zygomatic implant planning in general, and it can also be difficult to use correctly. This is due to the misalignment between the implant trajectory, identifiable with the cross-section, and the prosthetic axis of the abutment that defines the implant emergence. Furthermore, to know approximatively how much bone can be engaged in the different implant trajectories can influence the planning and the surgical choices. In addition, the approximative implant length required to choose a specific implant trajectory can facilitate the surgeon to choose a pool of implants for carrying out the intervention.

Therefore, the surgeon can consider the engageable bone by starting from an ideal occlusal emergence, and the implant trajectory can be chosen on the basis of the clinical case, utilizing either a more internal or external technique.

## Figures and Tables

**Table 1:** Measurements on different trajectories. SD = standard deviation. Lines 1, 2, and 3 are the internal, intermediate, and external trajectories, respectively, and all the planes are included; a, b, c indicate significant differences between lines 1, 2, and 3 for each variable (total length, bone engagement, intra-sinus path, extra-sinus path).

	Line	Mean (mm)	SD (mm)
Total length	1	61.82 ^a^	6.95
	2	53.14 ^b^	6.61
	3	46.36 ^c^	7.01
	Total	53.77	9.33
Bone engagement	1	20.03 ^a^	8.06
	2	19.00 ^b^	6.30
	3	14.72 ^c^	3.35
	Total	17.92	6.62
Intra-sinus path	1	13.28 ^a^	10.16
	2	4.65 ^b^	6.66
	3	0.03 ^c^	0.46
	Total	5.99	8.91
Extra-sinus path	1	28.51 ^a^	10.86
	2	29.48 ^b^	7.86
	3	31.60 ^c^	6.29
	Total	29.86	8.64

**Table 2:** Measurements on different planes. SD = standard deviation. All the lines are included. a, b, c indicate significant differences between plane 0, 4, 8, and 12 for each variable (total length, bone engagement, intra-sinus path, extra-sinus path).

	Plane (°)	Mean (mm)	SD (mm)
Total length	0	52.60^a^	9.53
	4	55.01^b^	9.99
	8	54.27^c^	9.07
	12	53.22^d^	8.50
	Total	53.77	9.33
Bone engagement	0	18.60 ^a^	6.18
	4	19.43 ^b^	6.44
	8	17.55 ^c^	6.64
	12	16.09^d^	6.74
	Total	17.92	6.62
Intra-sinus path	0	6.32^a^	9.50
	4	7.69^b^	9.96
	8	6.14^a^	8.73
	12	3.80^c^	6.65
	Total	5.99	8.91
Extra-sinus path	0	27.66^a^	9.10
	4	27.89^a^	9.16
	8	30.58^b^	7.79
	12	33.32^c^	7.11
	Total	29.86	8.64

**Table 3:** Bone engagement and intra-sinus path on different lines and planes to anterior and posterior emergences.

Implant Emergence	Plane (°)	Line	Bone Engagement (mm)	Intra-Sinus Path (mm)
Anterior	0	1	24.26 ± 5.84 19.02 ± 4.93 14.41 ± 2.92 19.23 ± 6.20	7.45 ± 7.61 3.28 ± 5.10 0.03 ± 0.44 3.59 ± 6.10
		2		
		3		
		Total		
	4	1	24.09 ± 6.36 20.18 ± 5.67 16.02 ± 2.98 20.10 ± 6.16	11.64 ± 7.89 5.08 ± 6.08 0.07 ± 0.68 5.60 ± 7.45
		2		
		3		
		Total		
	8	1	21.40 ± 6.91 20.84 ± 5.87 16.67 ± 2.61 19.63 ± 5.83	11.59 ± 7.62 4.52 ± 5.77 0.00 ± 0.00 5.37 ± 7.29
		2		
		3		
		Total		
	12	1	20.00 ± 7.46 21.29 ± 5.26 16.45 ± 2.43 19.25 ± 5.81	10.05 ± 7.07 3.51 ± 5.16 0.03 ± 0.38 4.53 ± 6.54
		2		
		3		
		Total		
Posterior	0	1	21.31 ± 6.38 17.98 ± 6.33 14.65 ± 3.04 17.98 ± 6.10	19.94 ± 10.91 7.19 ± 8.01 0.03 ± 0.43 9.05 ± 11.35
		2		
		3		
		Total		
	4	1	23.08 ± 6.92 18.23 ± 6.58 14.90 ± 2.92 18.74 ± 6.66	21.08 ± 10.30 8.24 ± 8.45 0.04 ± 0.55 9.79 ± 11.58
		2		
		3		
		Total		
	8	1	14.58 ± 7.88 18.40 ± 7.10 13.40 ± 3.31 15.46 ± 6.75	16.32 ± 10.27 4.32 ± 6.93 0.06 ± 0.61 6.90 ± 9.92
		2		
		3		
		Total		
	12	1	11.48 ± 6.54 15.99 ± 6.78 11.29 ± 3.04 12.92 ± 6.10	8.16 ± 8.98 1.07 ± 3.86 0.00 ± 0.00 3.07 ± 6.70
		2		
		3		
		Total		
